# Supplementary material for: Predicting Physician Consultations for Low Back Pain Using Claims Data and Population-Based Cohort Data—An Interpretable Machine Learning Approach
Source: Int J Environ Res Public Health. 2021 Nov 16;18(22):12013. doi: 10.3390/ijerph182212013 (PMC8622753; doi:10.3390/ijerph182212013)
Supplement: Supplementary file 1 [file ijerph-18-12013-s001.zip › ijerph-1406738-supplementary.pdf]

Supplement to the manuscript:

# *Predicting physician consultations for low back pain using claims data and population-based cohort data – an interpretable machine learning approach.*

Adrian Richter, Julia Truthmann, Jean François Chenot, Carsten Oliver Schmidt

## Table of Contents

List of Tables ........ 2
List of Figures ........ 2
List of ICD-10 codes ........ 3
List of physicians' fee schedules ........ 4
Methods ........ 5
  Distribution of the primary outcome ........ 5
  Candidate variables ........ 6
  Non-linear associations ........ 6
  Exploratory variable selection ........ 9
  Best subset ........ 11
  Model training algorithm ........ 12
  Scores for model calibration ........ 13
  Missing data ........ 14
    Imputation setting ........ 14
    Imputation diagnostics ........ 15
  Comparative analyses ........ 17
Results ........ 19
  Association of covariates ........ 19
  Best subset selection ........ 20
    Brier scores (Zero-part) ........ 20
    Dawid-Sebastiani scores (Count-part) ........ 22
  Goodness of fit ........ 24
  Prediction accuracy and missing data ........ 25
  Subgroup analyses ........ 26
References ........ 27

# List of Tables

Table S1: List of ICD-10 Codes associated with low back pain. ........ 3
Table S2: List of physicians' fee positions related to back pain ........ 4
Table S3: Results of a generalized additive model for nonlinear associations. ........ 6
Table S4: Candidate variables and results of explorative variable selection. ........ 10
Table S5: Matrix of all possible variable combinations. ........ 11
Table S6: Imputation diagnostics. ........ 15
Table S7: Subgroup analyses through stratification for seek of care. ........ 26

# List of Figures

Figure S1: Frequency of ICD-10 codes for back pain observed in the training data. ........ 5
Figure S2: Nonlinear associations of covariates with the presence of ICD-10 codes ........ 7
Figure S3: Distributional plots of all continuous candidate variables ........ 8
Figure S4: Scheme of the Validation algorithm to assess model prediction accuracy. ........ 12
Figure S5: Variable importance resulting from a tuned random forest approach ........ 18
Figure S6: Correlation plot of continuous or count data. ........ 19
Figure S7: Brier scores claims data. ........ 20
Figure S8: Brier scores SHIP data. ........ 21
Figure S9: Brier scores joined data. ........ 21
Figure S10: Dawid-Sebastiani scores claims data. ........ 22
Figure S11: Dawid-Sebastiani scores SHIP data. ........ 22
Figure S12: Dawid-Sebastiani scores joined data. ........ 23
Figure S13: Rootograms of the optimal model ........ 24
Figure S14: ROC curves for the best subset model with multiple imputations. ........ 25

## List of ICD-10 codes

The following list contains all ICD-10 codes used for the definition of low back pain.

*Table S1: List of ICD-10 Codes associated with low back pain.*

| ICD-10 codes | Code label |
|---|---|
| M40 | Kyphosis and lordosis |
| M41 | Scoliosis |
| M42 | Spinal osteochondrosis |
| M43 | Other deforming dorsopathies |
| M45 | Ankylosing spondylitis |
| M46 | Other inflammatory spondylopathies |
| M47 | Spondylosis |
| M48 | Other spondylopathies |
| M49 | Spondylopathies (not elsewhere classified) |
| M51 | Other intervertebral disc disorders |
| M53 | Other dorsopathies (not elsewhere classified) |
| M54 | Back pain (not elsewhere classified) |

We excluded all codes starting with 3-character ICD-10 codes *M50.XX,* which are used in cases of *cervical disc damages*. From the remaining ICD-10 codes, we have further removed:

*M42.12, M42.13, M42.92, M42.93, M43.02, M43.12, M43.22, M43.3, M43.4, M43.6, M47.12, M47.14, M47.21, M47.22, M47.23, M47.24, M47.82, M47.83, M47.84, M47.91, M47.92, M47.93, M47.94, M48.02, M48.03, M48.04, M50.-, M50.0, M50.1, M50.2, M50.3, M50.8, M50.9, M53.0, M53.1, M53.22, M53.23, M53.24, M53.82, M53.83, M53.92, M53.94, M54.01, M54.02, M54.03, M54.04, M54.11, M54.12, M54.13, M54.14, M54.82, M54.83, M54.84, M54.91, M54.92, M54.93, M54.94*

as they all related to cervical or thoracic diagnoses.

## List of physicians' fee schedules

The following list of the physicians’ fee schedule have been selected and divided into five groups.

*Table S2: List of physicians' fee positions related to back pain*

| Group | Codes | Label |
|---|---|---|
| General practitioner | 03000 | Specific code for treatment within standby |
| | 03003 | 19th to 54th year of age |
| | 03004 | 55th to 75th year of age |
| | 03005 | from 76th year of age |
| | 03111 | between 2005 - 2013: 6th to 59th year of age |
| | 03112 | between 2005 - 2013: from 60th year of age |
| Neurologist | 16211 | 6th to 59th year of age |
| | 16212 | from 60th year of age |
| | 16232 | Additional fee for diagnostic/treatment |
| Orthopedist | 18211 | 6th to 59th year of age |
| | 18212 | from 60th year of age |
| | 18331 | Additional fee for diagnostic/treatment |
| Interventions | 30201 | Manual therapy |
| | 30700, 30702 | Special pain therapy |
| | 30724, 34503, 30731, 02360 | Injection therapy |
| | 30790, 30791 | Acupuncture |
| | 02510 | Thermotherapy |
| | 02511 | Electrotherapy |
| Imaging | 34221, 34222 | X-ray |
| | 34223, 34311 | CT |
| | 34411 | MRI |

# Methods

## Distribution of the primary outcome

The primary outcome (number of ICD-10 codes for back pain) has a highly skewed distribution with a considerable quantity of zero's (Figure S1 below).

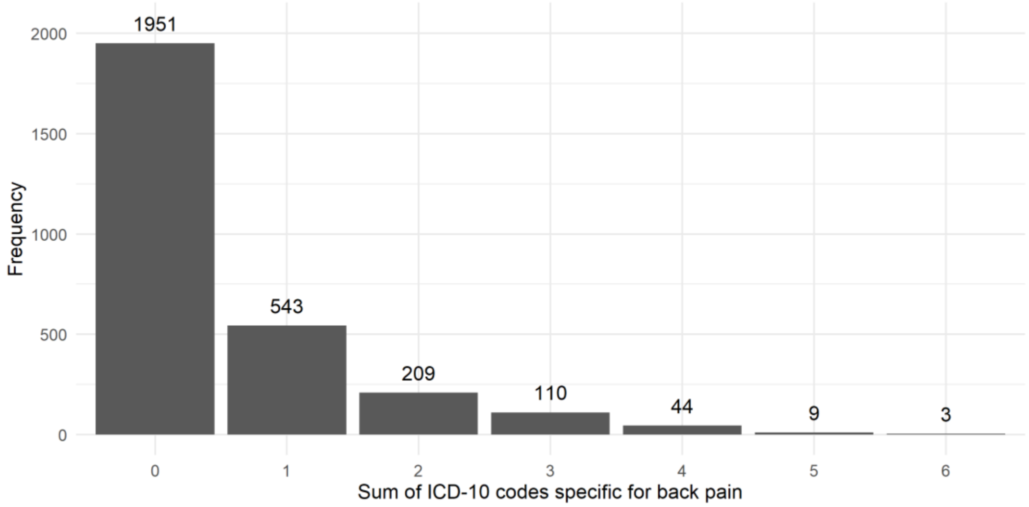


*Figure S1: Frequency of ICD-10 codes for back pain observed in the training data.*

The score test rejected the hypothesis of no zero inflation (p<<0.001).

```
## Score test for zero inflation
##
##     Chi-square = 661.56823
##     df = 1
##     pvalue: < 2.22e-16
```

## Candidate variables

We selected a broad sequence of variables from both data sources, which were presumably related to the frequency of low back pain episodes as indicated by ICD-10 codes. Table S4 in Exploratory variable selection shows the selected candidate variables after revision of univariate associations and interaction terms. In addition to these variables, we examined univariate associations of the following variables with the outcome:

- job type (e.g. farmer, freelancer)
- average von Korff back pain severity
- comorbidities: diabetes (yes vs. no), diabetes with complications (no vs. diabetes without complications vs. diabetes with complications), heart failure, atrial fibrillation, myocardial infarction, stroke, cancer
- interaction terms: PHQ-9 strata: sex.

Compared to the employment/workability status (Table S4), the job type was hardly associated with the outcome. Instead of the average of two von Korff items (back pain severity, impairment due to back pain), we used the original items in the modelling process. The above mentioned comorbidities (diabetes, cardiovascular disease, and cancer) origin from interviews of the SHIP participants and represent self-reported diseases. We summarized them into a single variable named *competing diseases* (none, one, >one of: diabetes, cardiovascular disease, and cancer) as all univariate coefficients indicated a negative association with the number of ICD-10 codes. Using claims data, we were able to compute the Charlson comorbidity index based on the approach from [1].

## Non-linear associations

The presence of non-linear associations was examined using a generalized additive model [2] provided by the R package *mgcv*. To discriminate nonlinear effects in the zero-part from those in the count-part we applied two types of models: (a) a binomial distribution for the zero-part and (b) negative binomial for the count-part.

*Table S3: Results of a generalized additive model for nonlinear associations.*

| Terms | edf | Ref.df | Chi.sq | p-value | Model |
|---|---|---|---|---|---|
| s(age) | 5.1 | 6.2 | 68.101 | 0.000 | Zero |
| s(bmi) | 2.2 | 2.8 | 5.485 | 0.153 | Zero |
| s(height) | 1.0 | 1.0 | 0.625 | 0.429 | Zero |
| s(weight) | 1.0 | 1.0 | 0.167 | 0.685 | Zero |
| s(sf12_pcs) | 2.2 | 2.8 | 19.183 | 0.000 | Zero |
| s(phq_sum_score) | 5.7 | 6.6 | 10.114 | 0.179 | Zero |
| s(house_income) | 2.2 | 2.8 | 22.143 | 0.000 | Zero |
| s(age) | 1.2 | 1.4 | 6.043 | 0.019 | Count |
| s(bmi) | 1.0 | 1.0 | 1.171 | 0.279 | Count |
| s(height) | 1.0 | 1.0 | 1.246 | 0.264 | Count |
| s(weight) | 1.0 | 1.0 | 1.123 | 0.289 | Count |
| s(sf12_pcs) | 1.0 | 1.0 | 9.613 | 0.002 | Count |
| s(phq_sum_score) | 1.2 | 1.3 | 0.041 | 0.909 | Count |
| s(house_income) | 1.0 | 1.0 | 0.985 | 0.321 | Count |

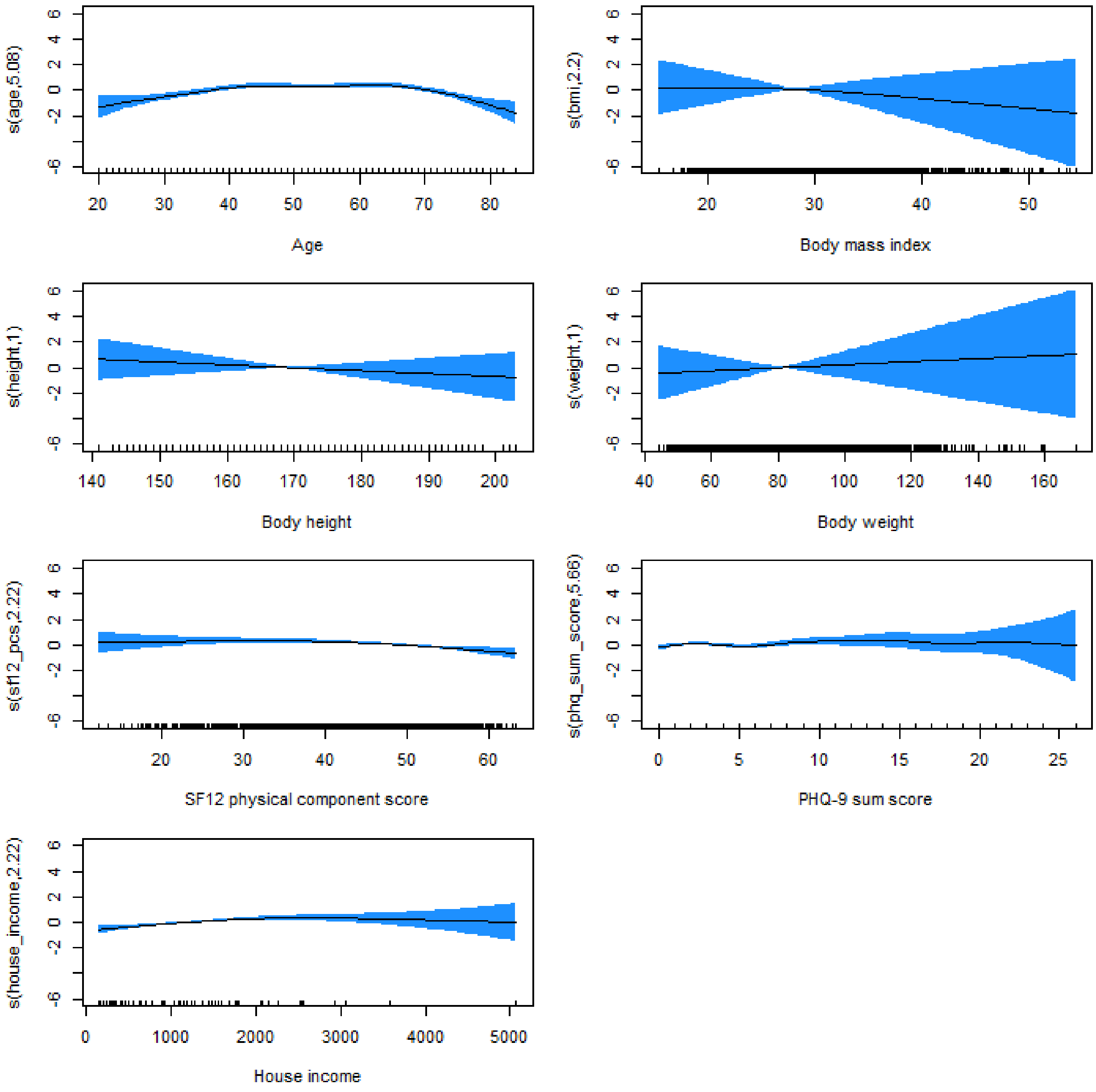


*Figure S2: Nonlinear associations of covariates with the presence of ICD-10 codes for back pain (yes/no).*

The results from this model suggest non-linear associations in the zero-part of the model for age, SF12 PCS, PHQ-9 sum score, and house income. Regarding the count-part none of the candidate variables appears to have non-linear associations, as effective degrees of freedom are all close to 1.

There are some caveats: the household income in the SHIP data represents a transformed variable. Participants of the SHIP study were asked about their income in non-equidistant

categories [([0; 500), [500; 900), [900; 1300), [1300; 1800), [1800; 2300), [2300; 2800), [2800; 3300), [3300; 3800), 3800; Inf)). These categories were then transformed along the interval means and standardized according the number of individuals living in the respective household ($\sqrt{\# \, individuals}$). The respective values appear to be continuous, but base in fact on categorical values. Please see also Figure S4 below where we made use of the R package *dataquieR* to illustrate possible outliers [3]. We therefore decided to not model a nonlinear functional form (spline) of this variable.

In summary, based on AIC information criteria, we considered restricted natural splines for age (degrees of freedom df=4) and SF12 physical component scale (df=2) as candidate variables.

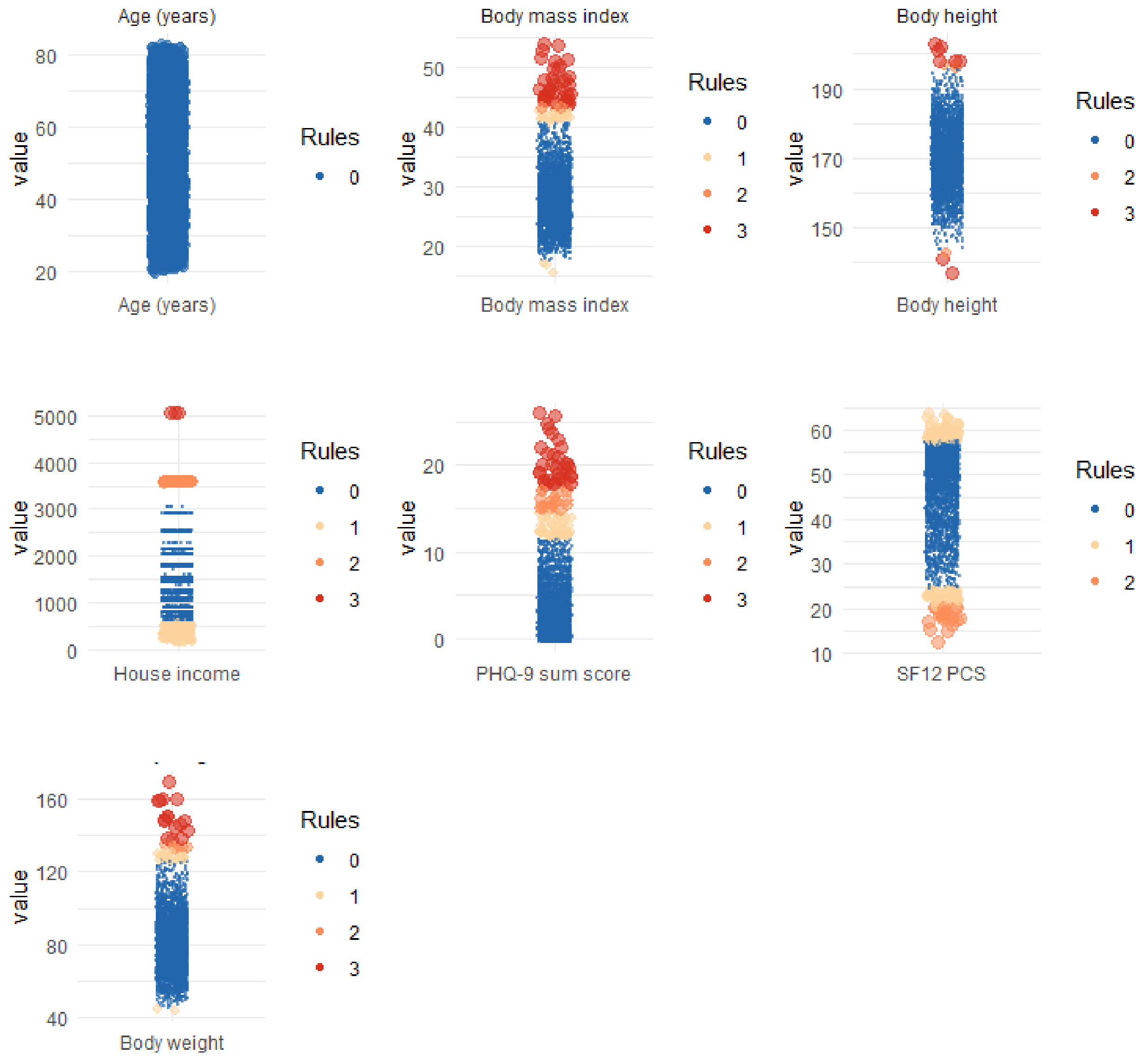


*Figure S3: Distributional plots of all continuous candidate variables and the classification of outliers. Please see this website for annotation of the different outlier classification rules.*

**Exploratory variable selection**

Best subset selection is computationally expensive with $2^p - 1$ possible combinations of covariates. To minimize computational time, we applied *model-based boosting* (MBB) to conduct an explorative variable selection for the SHIP data. MBB is applicable in high-dimensional settings (n<<p) and in the presence of multi-collinearity [4]. The latter is of particular interest for our approach since we examined some variables in alternative forms, e.g. age (original, discretized, smoothed).

In brief, MBB starts from an intercept model in which each *base-learner* or candidate variable is iteratively evaluated in terms of fitting a loss function (negative log-likelihood) best. In each iteration only the best base-learner, with respect to minimizing residual sum of squares, will be used to update the model [4]. The R package mboost [5] has been used to build the MBB model in 2000 iterations (maximum mstop). Usually the best model is then identified via resampling. However, we applied stability selection [6] after model building to select covariates being predictive in 20% of the resampled data, i.e. contributing to the model in at least 20% of resampled data. This setting is less restrictive, i.e. more variables are retained, recommendations suggest to choose a threshold of 50% or higher [6]. However, we pursued an initial set of promising candidate variables and to remove competing effect definitions, we omitted the evaluation of the optimal MBB model. All predictors as specified in Table S4 were examined as candidate learners.

In case of alternative effect definition, e.g. regarding age, we chose the effect with highest selection frequency in MBB.

*Table S4: Candidate variables and results of explorative variable selection.*

| Characteristic | Data type and if required categories and explanation | Model part | Selection frequency ZERO | Selection frequency COUNT |
|---|---|---|---|---|
| Sex | female \| male | zero | 0.68 | 0.00 |
| Age | integer | count | 0.00 | 0.61 |
| Age (categorized) | [20; 40); [40; 70); [70;Inf): age has been categorized based on clinical hypotheses | zero | 1.00 | 0.55 |
| Age (spline) | B-splines with 5 degrees of freedom (knots at quintiles) | | 0.10 | n.a. |
| Use of opioids | no \| yes | count | 0.03 | 0.38 |
| Use of benzodiazepine | no \| yes | | 0.01 | 0.01 |
| Use of NSAR | no \| yes; self-reported use of NSAR (over-the-counter-drug) | | 0.10 | 0.11 |
| Use of antidepressants | no \| yes | | 0.00 | 0.04 |
| No. of drugs used in last 7d | integer | count | 0.00 | 0.71 |
| Family status | single \| married \| divorced \| widowed | zero, count | 0.57 | 0.27 |
| BMI | float | | 0.00 | 0.02 |
| Back pain in last 3 month (NRS) | integer | zero, count | 0.99 | 0.41 |
| Impairment by back pain in last 3 month (NRS) | integer | count | 0.03 | 0.99 |
| PHQ-9 | Sum score | | 0.00 | 0.00 |
| SF12 Physical component scale | float | | 0.00 | 0.16 |
| SF12 Physical component scale (spline) | B-splines with 2 degrees of freedom (knot at median) | | 0.08 | n.a. |
| Sport activities | <1h a week \| 1-2h a week \| > 2h a week | count | 0.03 | 0.35 |
| Physician visits (last year) | None \| GP only \| Specialist only \| GP and specialist (visits can be unrelated to back pain) | zero, count | 1.00 | 0.73 |
| Body height | float | | 0.00 | 0.01 |
| Body weight | float | | 0.03 | 0.02 |
| Household income | float | zero | 0.85 | 0.09 |
| School years | <10 \| 10 \| >10 | count | 0.06 | 0.20 |
| Physical demanding job | no \| yes | | 0.04 | 0.04 |
| Workability status | employable \| retired \| unemployed | count | 0.00 | 0.34 |
| Competing diseases | 0 = None \| 1 = one \| 2 = >1 (of diabetes, cardiovascular disease, cancer) | zero | 0.21 | 0.09 |
| Depression (self-reported) | no \| yes | | 0.00 | 0.08 |
| Inflammatory joint disease | no \| yes | | 0.04 | 0.01 |
| Osteoarthritis | no \| yes | zero, count | 0.57 | 0.28 |
| Disc prolapse | no \| yes | zero, count | 0.96 | 0.81 |
| Pressure pain (related to back pain) | no \| yes | zero | 0.31 | 0.01 |
| Dysesthesia (tingling, related to back pain) | no \| yes | zero, count | 0.43 | 0.38 |
| Radiating back pain | 0 = none \| 1 = yes, gluteal only \| 2 = yes, to knee \| 3 = yes, to lower leg | zero, count | 0.53 | 0.41 |
| Interactions: | | | | |
| depression: sex | | | 0.08 | 0.04 |
| depression: PHQ-9 | | | 0.06 | 0.15 |
| disc prolapse: radiating back pain | | zero, count | 0.22 | 0.63 |

*n.a.: not applicable as non-linear associations were not necessary to model the count part of the hurdle model. All variables marked with a gray background were removed by model-based boosting under stability selection.

**Best subset**

From a vector of candidate variables (in the following example A, B, C) we specified a matrix of all their possible combinations in the following manner:

```r
# Variables: A, B, and C
covars <- c("A", "B", "C")

# each of these variables can be selected (1) or not (0)
ps <- rep(list(0:1), length(covars))

# create all possible combinations
ps_claims <- expand.grid(ps)

# remove row in which all variables are "Null"
ps_claims <- ps_claims[-1, ]
names(ps_claims) <- covars
```

*Table S5: Matrix of all possible variable combinations.*

| A | B | C |
|---|---|---|
| 1 | 0 | 0 |
| 0 | 1 | 0 |
| 1 | 1 | 0 |
| 0 | 0 | 1 |
| 1 | 0 | 1 |
| 0 | 1 | 1 |
| 1 | 1 | 1 |

The validation algorithm iterated in each bootstrap sample over the rows of this matrix and selected the model-formula according to binary values in this matrix (1 = *TRUE*).

## Model training algorithm

Model training and evaluation were conducted in separate data to avoid overfitting [7]. In absence of external (independent) validation data, a so-called internal validation of a model should be applied, i.e. using a subsample of the same data source, which is not involved in model training. This approach is inferior to an external validation but a crucial model evaluation step [8].

We adapted a common approach of model validation as described by Filzmoser et. al [8]. Therefore, the SHIP-TREND-0 data were randomly splitted into *training-data* and *validation-data* in a ratio of 3:1 (n=2869:968). Figure S4 shows the scheme of this approach:

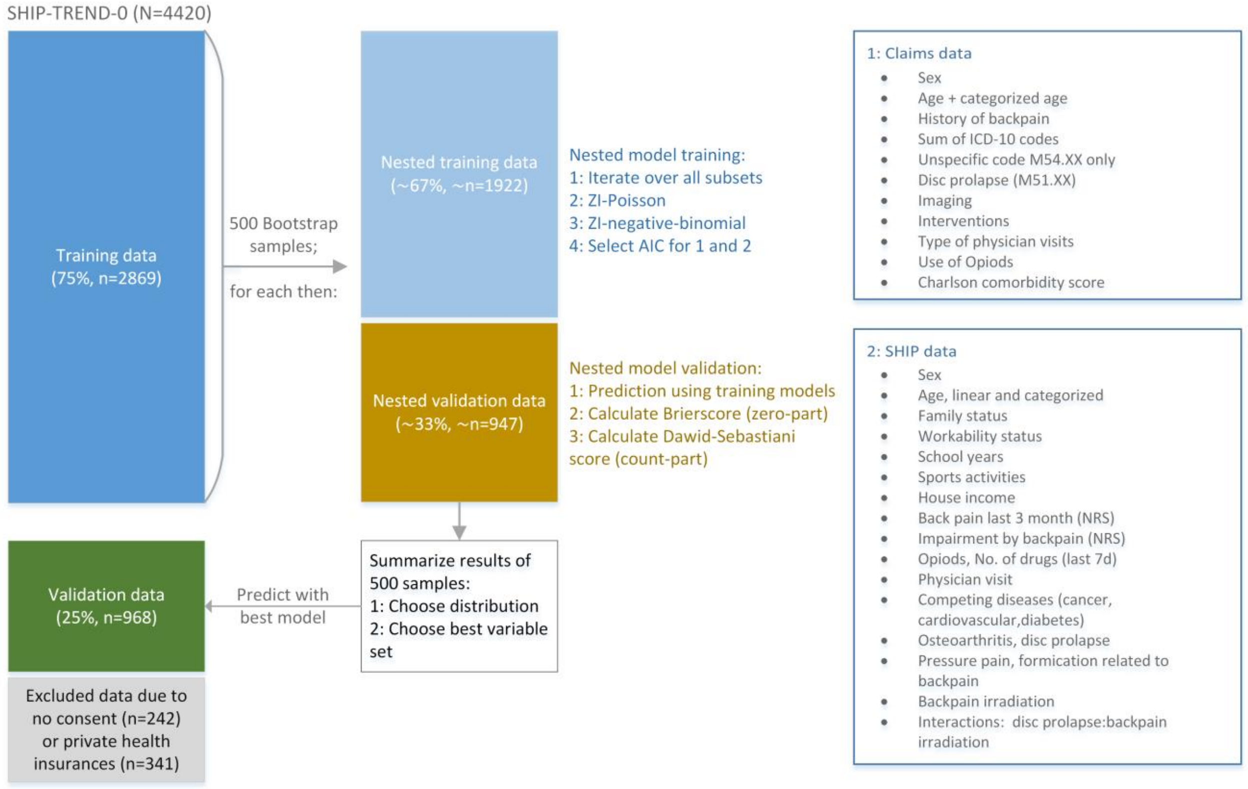


*Figure S4: Scheme of the Validation algorithm to assess model prediction accuracy.*

In each step of the nested model training we iterated over a matrix of predictors (please see Best subset for explanation) and conducted zero-inflated *Poisson* and negative-binomial count data models for all possible combinations of covariates. Respective information criteria (AIC) have been saved in each iteration to choose the most appropriate distribution type after completion of the nested model training. Each model result was used to predict the outcome of interest in the nested validation data.

The accuracy of the nested prediction performance has been evaluated using the strictly proper scoring rules of *Brier score* and the *Dawid-Sebastiani score* [10–12]. The calculation and notation

of these scores is mentioned under Scores for model calibration and has been applied using the R package *surveillance* [13].

Overall, this scheme was applied three times:

- 1st for the claims data
- 2nd for the SHIP data
- 3rd for the joined data using only the best subset from either claims and SHIP.

Due to the computational complexity, e.g. 8.191.000 models ($2^{13}$- 1 subsets * 500 samples * 2 distributions) were calculated for the claims data alone, we used the R package *doParallel* [14] to apply parallel computations. Further, we used the high-performance-cluster provided by the computation center of the University of Greifswald [15].

**Scores for model calibration**

We used two strictly proper scoring rules to evaluate a model's prediction accuracy. For the *zero-part* of the zero-inflated count data regression we used the *Brier score* [16] and for the *count-part* the *Dawid-Sebastiani score* [11] proposed by [10]. The *Brierscore* has the following notation:

$$BS = \frac{1}{N}\sum_{i=1}^{N}(p_i - o_i)^2.$$

where $p_i$ is the predicted probability for individual $i$ and $o_i$ the observed event of individual $i$.

The *Dawid-Sebastiani score* has the following notation for a negative-binomial [11] count data regression:

$$DSS_{(NBB)} = \frac{1}{N}\sum_{i=1}^{N}\left(\frac{(y_i-\mu_i)^2}{\mu_i(1+\frac{\mu_i}{\theta})} + log(\mu_i(1 + \frac{\mu_i}{\theta}))\right).$$

and in terms of a Poisson distribution with $\mu = \sigma^2 = \lambda$:

$$DSS_{(Poi)} = \frac{1}{N}\sum_{i=1}^{N}\left(\frac{(y_i-\lambda_i)^2}{\lambda_i} + log(\lambda_i)\right).$$

The calculation of these scores is implemented in the R package *surveillance* [12].

## Missing data

### *Imputation setting*

The overall frequency of missing values was low (<5%). Nevertheless, we first applied multiple imputations using chained equations provided by the R package *mice* ([17]). The imputation was conducted independently for training data and validation data.

Transformed variables, such as body mass index and strata of the PHQ score, were not imputed; instead passive imputation has been used, which was defined as:

```
# mice::methods for imputation
meths["bmi"] <- "~I(weight/((height/100)^2))"

# a customized function to categorize phq score into strata
mycutfun <- function(x) {
 x <- cut(x,
          right = FALSE,
          breaks = c(0, 1, 5, 10, 15, Inf),
          labels = c("no signs", "minimal", "mild", "moderate", "severe"))

}

# mice::methods for imputation
meths["phq_strata"] <- "~I(mycutfun(phq_sum_score))"
```

*Imputation diagnostics*

We used inbuilt functions of the R package mice for imputation diagnostics. Further, variance increase (%) by using Rubins' rule [18] for calculating the pooled variance from the variance between and within the imputed data sets.

Overall, the increase in variance was very low, for most covariates <1%. Due the small increase in variance and in line with recommendations for the application of multiple imputations in case of missingness < 5% [19] we restricted the imputations to $m = 1$ in the training data.

However, the validation data contained m=20 imputations.

*Table S6: Imputation diagnostics.*

| Variable | Category | Location | Before imputations | After imputations | V increase |
|---|---|---|---|---|---|
| Household income | | Mean | 1,296.77 | 1,297.83 | 4.58 |
| BMI | | Mean | 28.28 | 28.29 | 0.38 |
| Body weight | | Mean | 81.26 | 81.27 | 0.12 |
| Body height | | Mean | 169.37 | 169.37 | 0.28 |
| Back pain (NRS) | | Mean | 2.72 | 2.73 | 0.28 |
| Impairment due to back pain (NRS) | | Mean | 1.05 | 1.06 | 0.38 |
| SF12 PCS | | Mean | 46.97 | 46.90 | 1.24 |
| PHQ-9 sum score | | Mean | 3.97 | 3.97 | 3.74 |
| Use of medication (last 7d) | | Mean | 2.49 | 2.49 | 0.46 |
| Family status | Single | Proportion | 0.10 | 0.10 | 0.00 |
| | Married/Partner | Proportion | 0.77 | 0.78 | 0.00 |
| | Separated | Proportion | 0.06 | 0.06 | 0.00 |
| | Widowed | Proportion | 0.06 | 0.06 | 0.00 |
| Work categories | Never working | Proportion | 0.01 | 0.01 | 0.00 |
| | At desktop, not physically | Proportion | 0.30 | 0.30 | 0.02 |
| | At desktop and physically demanding | Proportion | 0.13 | 0.14 | 0.02 |
| | Not at desktop, not physically | Proportion | 0.20 | 0.22 | 0.11 |
| | Not at desktop but physically demanding | Proportion | 0.32 | 0.34 | 0.14 |
| Physical demanding job | yes | Proportion | 0.45 | 0.47 | 0.16 |
| | no | Proportion | 0.51 | 0.53 | 0.16 |
| Work status | employable | Proportion | 0.54 | 0.55 | 0.01 |
| | retired | Proportion | 0.32 | 0.36 | 0.00 |
| | unemployed | Proportion | 0.09 | 0.10 | 0.01 |
| School years | <10 | Proportion | 0.25 | 0.25 | 0.00 |
| | 10 | Proportion | 0.53 | 0.53 | 0.00 |
| | >10 | Proportion | 0.22 | 0.22 | 0.00 |
| Physical activity | No sport | Proportion | 0.22 | 0.22 | 0.01 |
| | 1-2h/week | Proportion | 0.57 | 0.58 | 0.01 |
| | >2h/week | Proportion | 0.20 | 0.20 | 0.00 |
| Radiating back pain | no | Proportion | 0.68 | 0.68 | 0.00 |
| | gluteal only | Proportion | 0.16 | 0.16 | 0.00 |
| | to knee | Proportion | 0.10 | 0.10 | 0.00 |
| | to lower leg | Proportion | 0.06 | 0.06 | 0.00 |
| Pressure pain | yes | Proportion | 0.17 | 0.17 | 0.01 |
| | no | Proportion | 0.82 | 0.83 | 0.01 |
| NSAR | no | Proportion | 0.90 | 0.90 | 0.00 |
| | yes | Proportion | 0.10 | 0.10 | 0.00 |
| Tingling, prickling | yes | Proportion | 0.12 | 0.12 | 0.00 |
| | no | Proportion | 0.88 | 0.88 | 0.00 |
| Claudication | yes | Proportion | 0.02 | 0.02 | 0.08 |
| | no | Proportion | 0.95 | 0.98 | 0.08 |
| Osteoarthritis | yes | Proportion | 0.29 | 0.30 | 0.01 |

| Variable | Category | Location | Before imputations | After imputations | V increase |
|---|---|---|---|---|---|
| | no | Proportion | 0.69 | 0.70 | 0.01 |
| Disc prolapse | yes | Proportion | 0.09 | 0.09 | 0.00 |
| | no | Proportion | 0.90 | 0.91 | 0.00 |
| Inflammatory joint disease | yes | Proportion | 0.05 | 0.06 | 0.01 |
| | no | Proportion | 0.93 | 0.94 | 0.01 |
| Competing disease | None | Proportion | 0.79 | 0.79 | 0.00 |
| | One | Proportion | 0.16 | 0.16 | 0.00 |
| | >1 | Proportion | 0.04 | 0.04 | 0.00 |
| Physician visit | No physician visit | Proportion | 0.18 | 0.19 | 0.01 |
| | GP visit only | Proportion | 0.56 | 0.56 | 0.01 |
| | Specialist only | Proportion | 0.03 | 0.03 | 0.01 |
| | GP and specialist | Proportion | 0.21 | 0.21 | 0.01 |
| Use of medication (last 7d, y/n) | yes | Proportion | 0.71 | 0.71 | 0.00 |
| | no | Proportion | 0.28 | 0.29 | 0.00 |
| Depression | no | Proportion | 0.69 | 0.71 | 0.02 |
| | yes | Proportion | 0.28 | 0.29 | 0.02 |
| Opioids | no | Proportion | 0.98 | 0.98 | 0.00 |
| | yes | Proportion | 0.02 | 0.02 | 0.00 |
| Benzodiazepine | no | Proportion | 0.98 | 0.98 | 0.00 |
| | yes | Proportion | 0.01 | 0.01 | 0.00 |
| Antidepressants | no | Proportion | 0.94 | 0.94 | 0.00 |
| | yes | Proportion | 0.06 | 0.06 | 0.00 |

**Comparative analyses**

For comparative analysis we applied *randomforest* (RF) in this study, which has been shown to provide excellent prediction accuracy [20,21]. In RF, bootstraps of original data, i.e. random samples of the data with replacement, are used to build ensembles of uncorrelated decision trees on so-called in-bag observations.

It is advised to build a high number of trees (*ntree*) in RF but the number of trees is not considered a tuning parameter [22]. The following parameters have a higher impact on prediction accuracy:

- the number of variables (*mtry*) considered for splitting the trees
- the minimum terminal node size (*nodesize*), and
- the sample size of inner bootstraps (*sampsize*).

We used the R package *tuneRanger* to conduct this tuning [22].

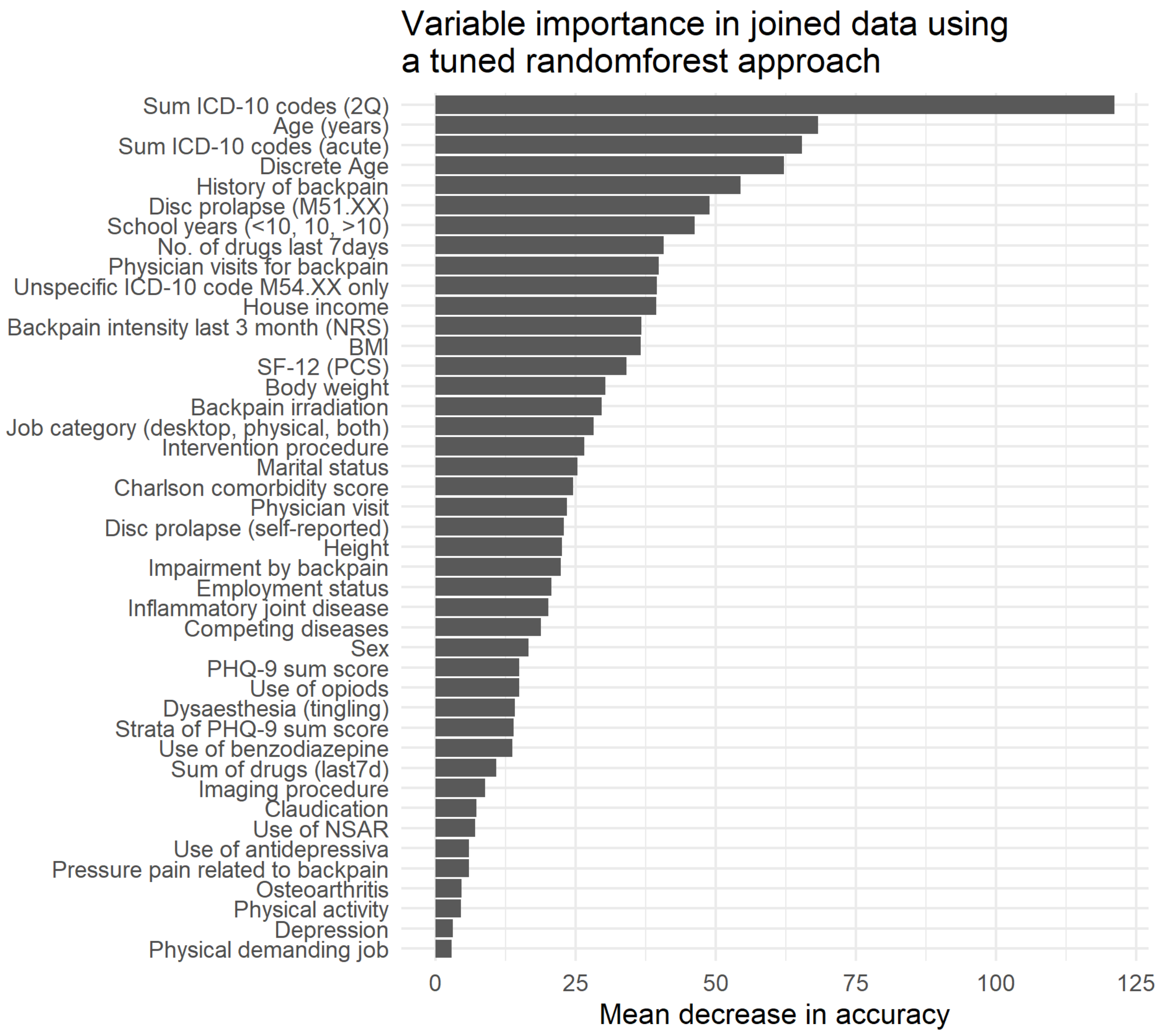


*Figure S5: Joined data means claims data and SHIP data analyzed together. Variable importance resulting from a tuned random forest approach (mtry=9, nodesize=60, sampsize = 1674, ntree=10000).*

## Results

### Association of covariates

For most continuous or count variables we found low to moderate associations. Particularly the association of self-reported back pain (SHIP-data) with the frequency of ICD-10 codes for back pain (claims data) was low.

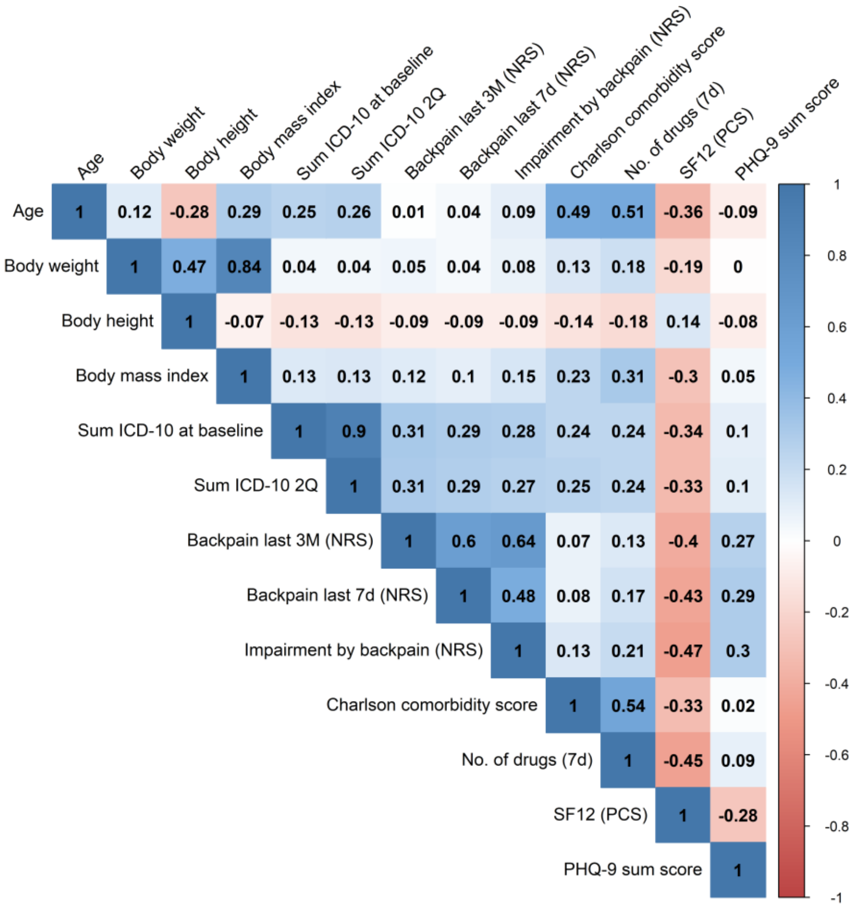


*Figure S6: Correlation plot of continuous or count data.*

## Best subset selection

In this section, the distribution of mean scores across all 500 bootstrap samples is shown for the individual candidate variable, the full set and the best subset of all candidate variables. The scores obtained for the models in the claims data are much lower on average, i.e. the predictive value of these variables is higher than those for the SHIP data.

Computational times for best subset were 28h for claims and SHIP data on two nodes à 16 cores, and 21h for joined best subsets on one node à 16 cores.

### *Brier scores (Zero-part)*

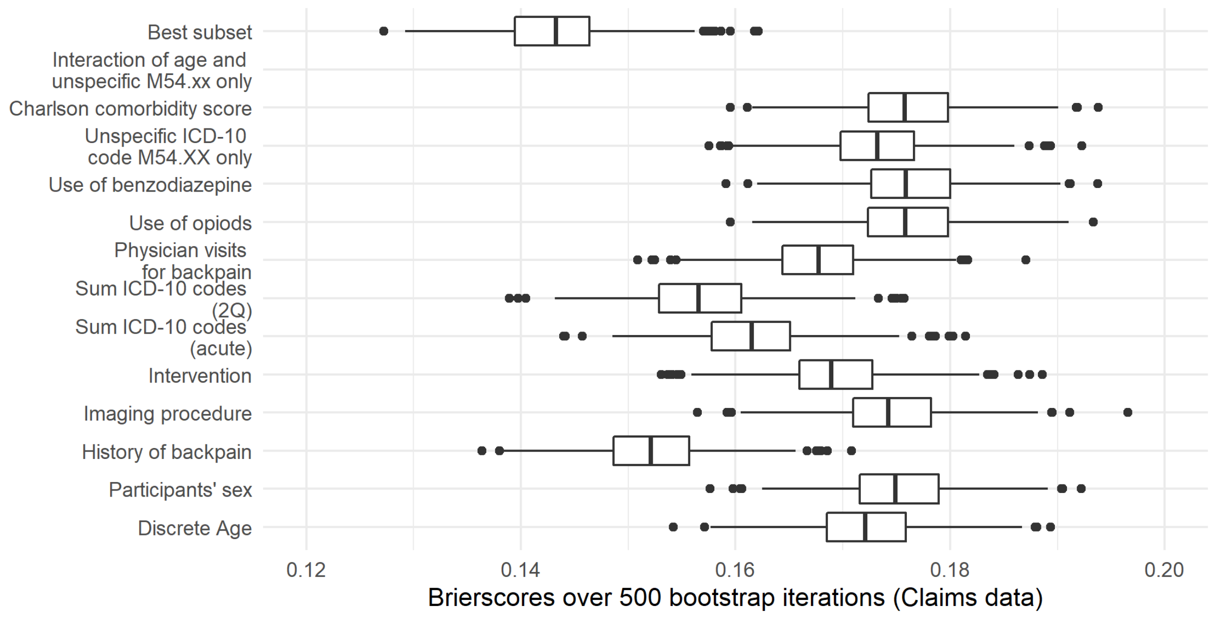


*Figure S7: Brier scores claims data.*

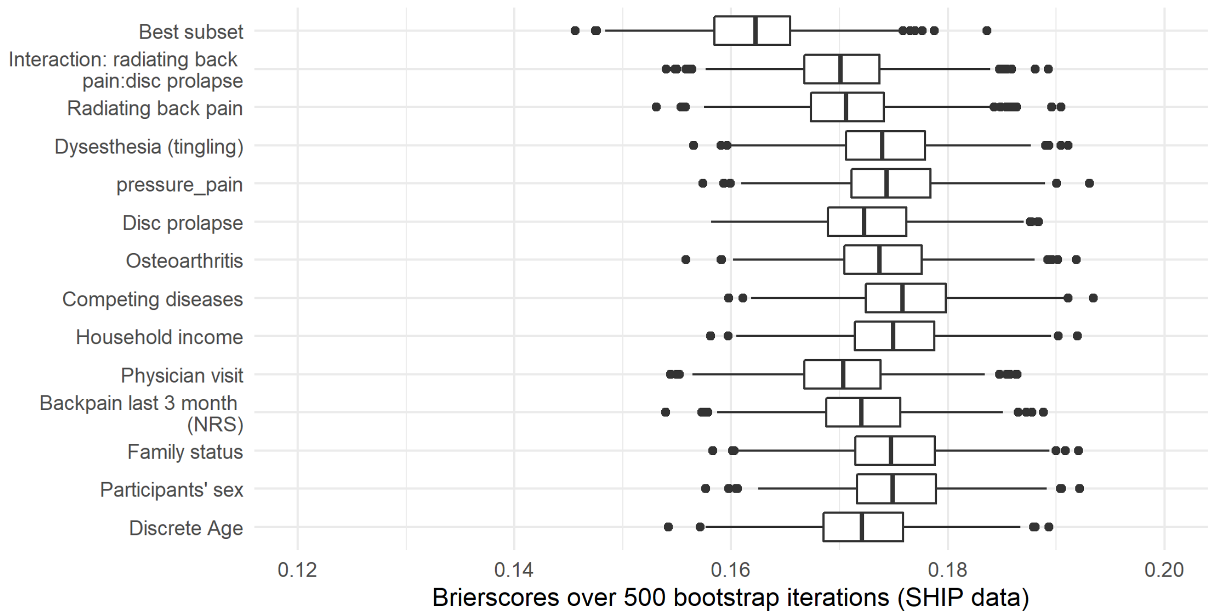


*Figure S8: Brier scores SHIP data.*

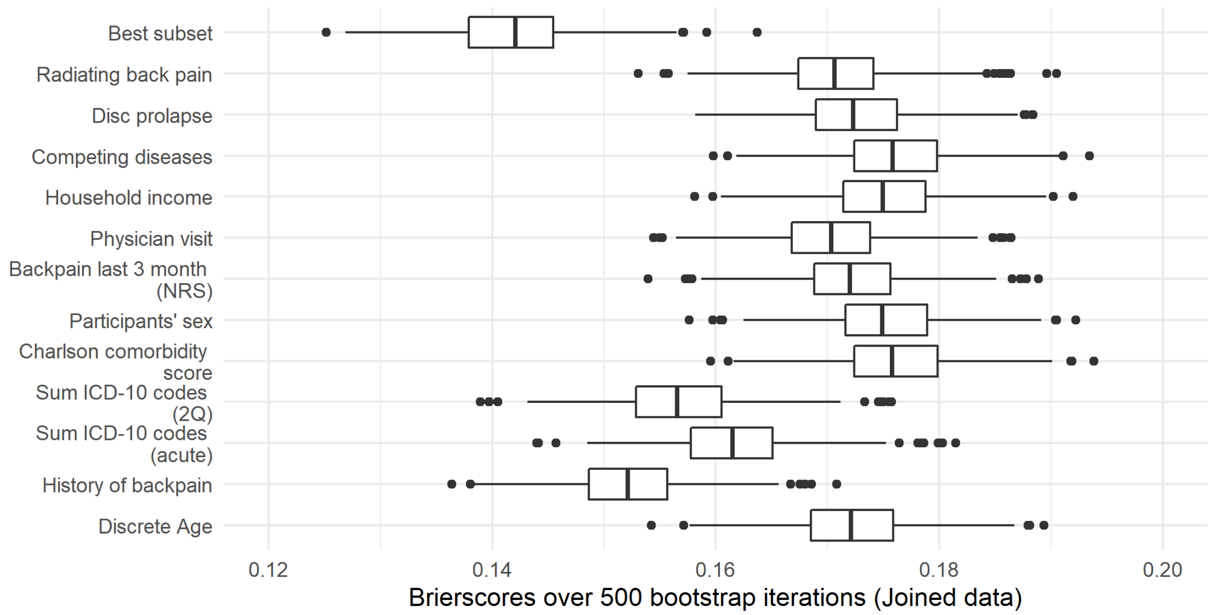


*Figure S9: Brier scores joined data.*

### ***Dawid-Sebastiani scores (Count-part)***

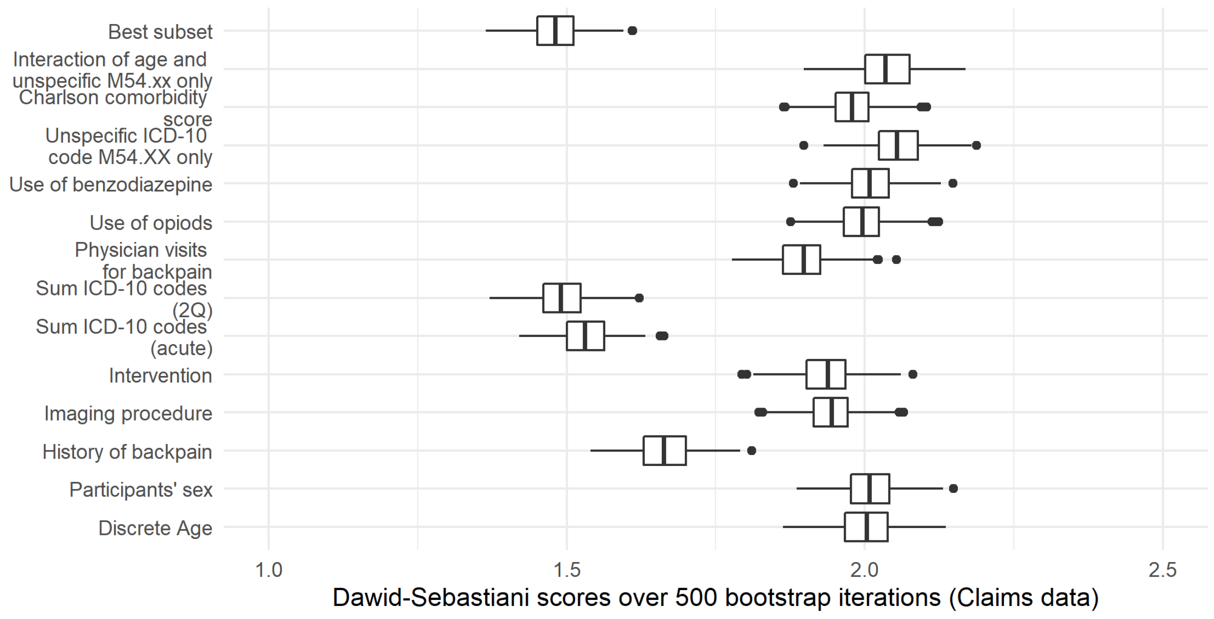


*Figure S10: Dawid-Sebastiani scores claims data.*

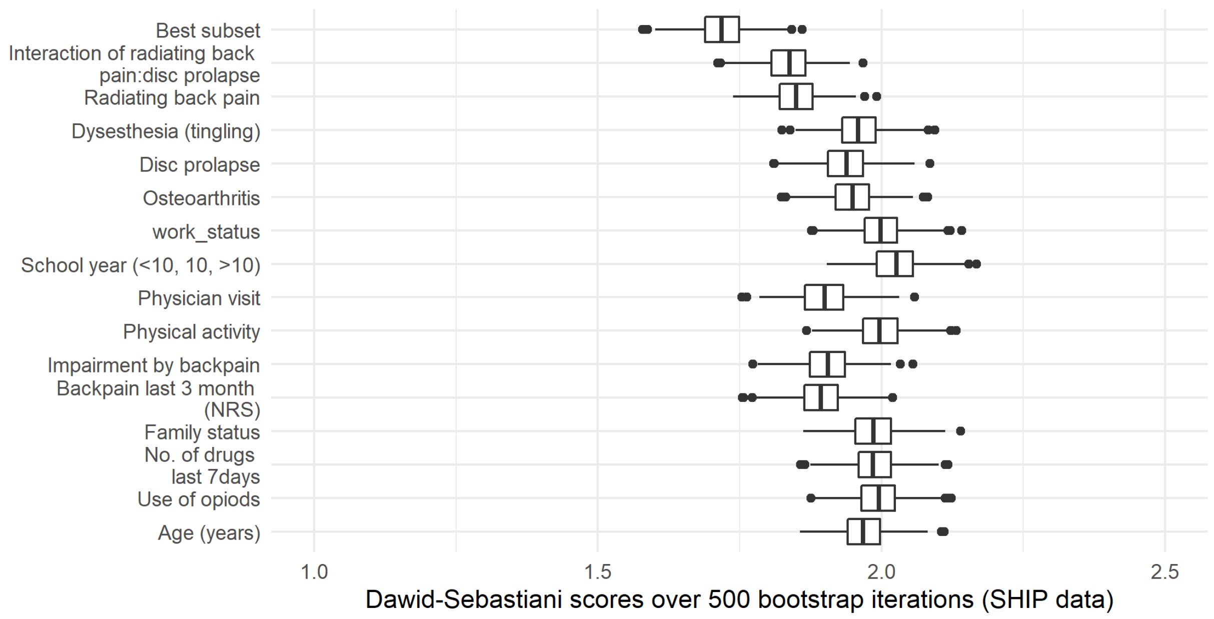


*Figure S11: Dawid-Sebastiani scores SHIP data.*

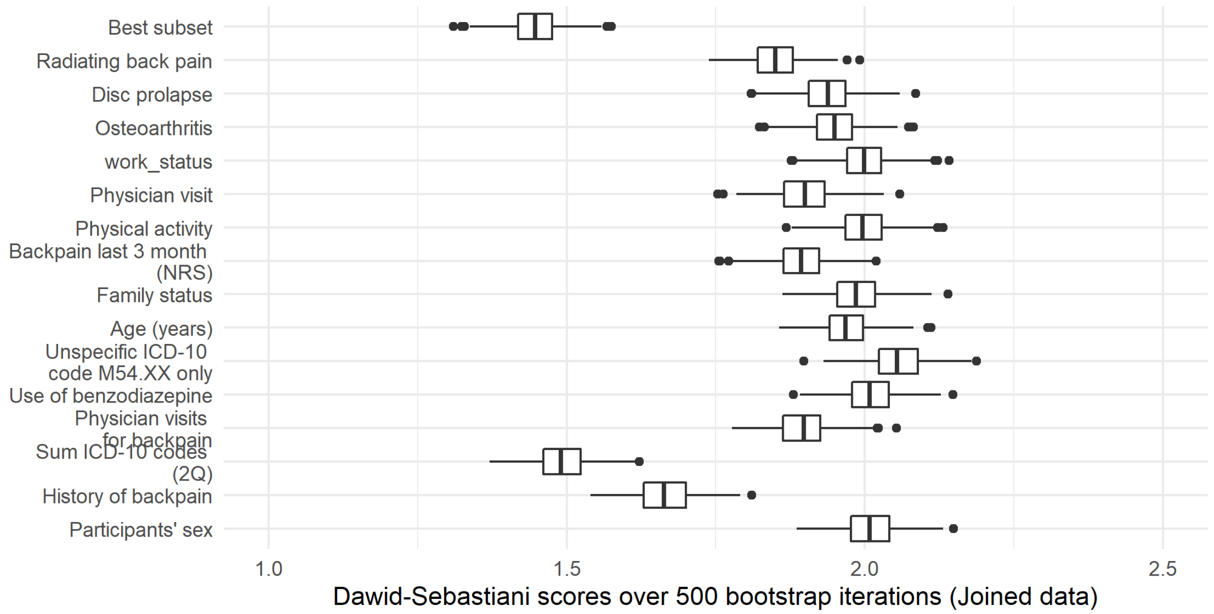


*Figure S12: Dawid-Sebastiani scores joined data.*

**Goodness of fit**

To visualize the fit of the optimal model obtained from the joined data we used rootograms [23]. The bars in Figure S13 correspond to the observed counts of ICD-10 codes. The red line indicates the fitted response of the hurdle model from a Poisson distribution.

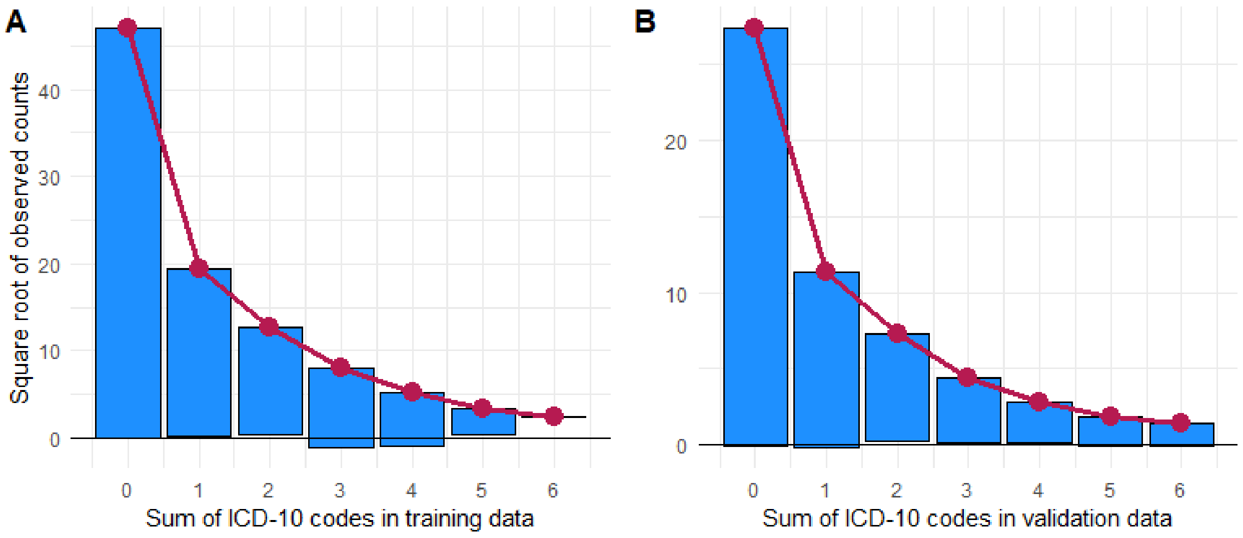


*Figure S13: Rootograms of the optimal model from the joined data evaluated in training data and the validation data.*

By design, the counts of the zero-part are perfectly fitted in the training data and slightly overestimated in the validation data, i.e. in the validation data the actual counts are slightly lower than what has been estimated by the model. The model fit is very reasonable.

**Prediction accuracy and missing data**

In Figure S14 we have depicted the ROC curves of predictions in the validation data for each imputed data set (m=20).

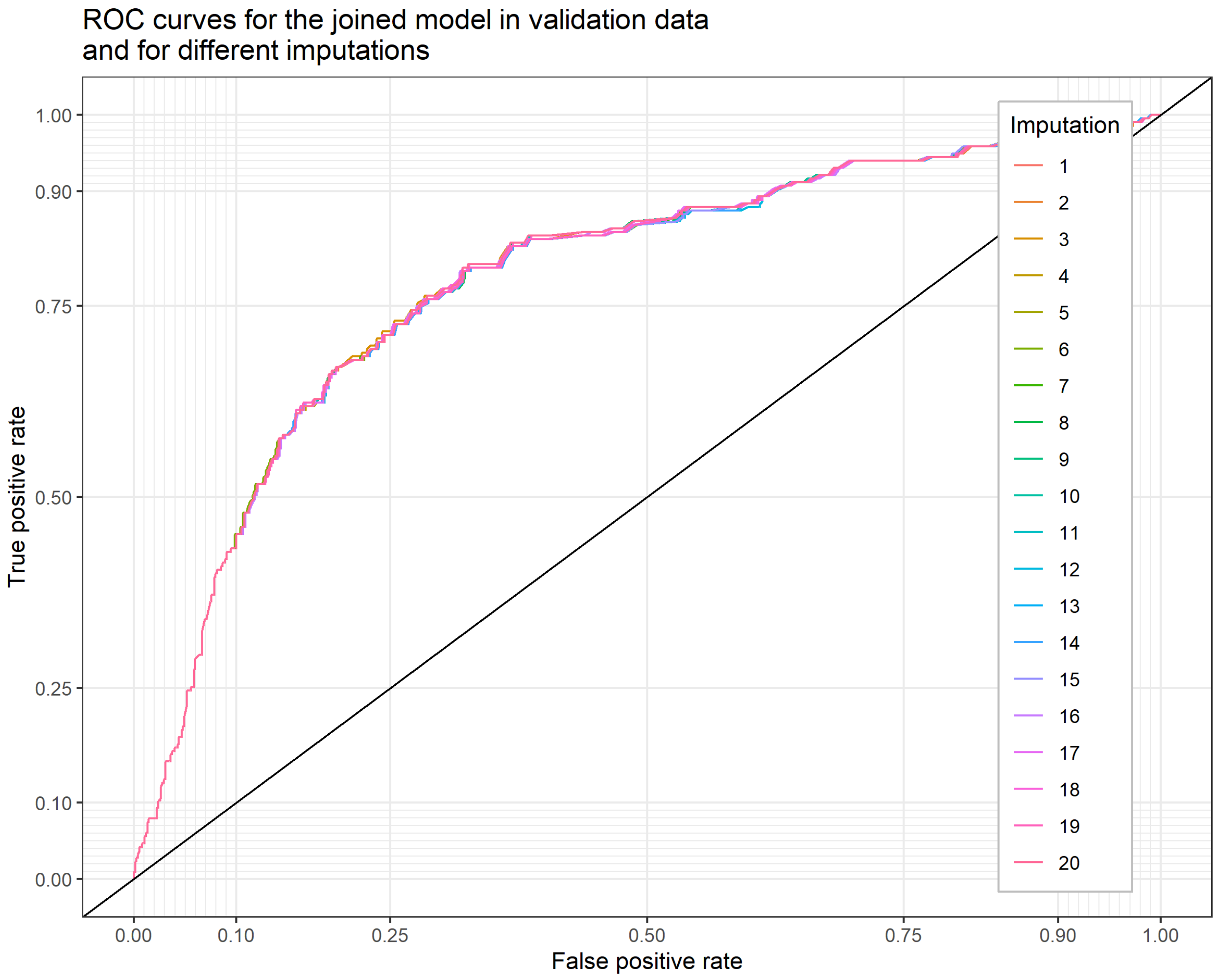


*Figure S14: ROC curves for the best subset model applied on validation data with multiple imputations.*

There is almost no difference between the different data sets which relates (a) to the low magnitude of missing data in most covariates and (b) that particularly in those variables selected as the best subset missingness was <1%.

## Subgroup analyses

*Table S7: Subgroup analyses through stratification for seek of care.*

| | Seek of care for low back pain* | | | |
|---|---|---|---|---|
| Characteristic | 0\|0 | 0\|1 | 1\|1 | 1\|0 |
| **N** | 2229 | 267 | 601 | 740 |
| **Age (years)** | | | | |
| Mean (SD) | 49.9 (16.0) | 49.8 (13.1) | 56.3 (12.3) | 58.7 (15.2) |
| **Age discrete** | | | | |
| <40 years | 670 (30.1%) | 64 (24.0%) | 58 (9.7%) | 102 (13.8%) |
| 40 to 69 years | 1239 (55.6%) | 186 (69.7%) | 448 (74.5%) | 419 (56.6%) |
| > 69 years | 320 (14.4%) | 17 (6.4%) | 95 (15.8%) | 219 (29.6%) |
| **Sex** | | | | |
| male | 1142 (51.2%) | 110 (41.2%) | 247 (41.1%) | 316 (42.7%) |
| female | 1087 (48.8%) | 157 (58.8%) | 354 (58.9%) | 424 (57.3%) |
| **Workability status** | | | | |
| Employable | 1333 (59.8%) | 174 (65.2%) | 301 (50.1%) | 298 (40.3%) |
| Retired | 663 (29.7%) | 66 (24.7%) | 253 (42.1%) | 378 (51.1%) |
| Unemployed | 233 (10.5%) | 27 (10.1%) | 47 (7.8%) | 64 (8.6%) |
| **Physical demanding job** | | | | |
| Yes | 1005 (45.1%) | 120 (44.9%) | 285 (47.4%) | 400 (54.1%) |
| No | 1224 (54.9%) | 147 (55.1%) | 316 (52.6%) | 340 (45.9%) |
| **Household income** | | | | |
| Mean (SD) | 1290 (656) | 1410 (707) | 1400 (632) | 1200 (538) |
| Missing | 1100 [167, 5070] | 1450 [149, 3580] | 1360 [149, 3580] | 1100 [167, 3580] |
| **Competing diseases** | | | | |
| None | 1815 (81.4%) | 229 (85.8%) | 470 (78.2%) | 514 (69.5%) |
| One | 335 (15.0%) | 32 (12.0%) | 115 (19.1%) | 171 (23.1%) |
| >One | 79 (3.5%) | 6 (2.2%) | 16 (2.7%) | 55 (7.4%) |
| **Inflammatory joint disease** | | | | |
| Yes | 77 (3.5%) | 10 (3.7%) | 56 (9.3%) | 66 (8.9%) |
| No | 2152 (96.5%) | 257 (96.3%) | 545 (90.7%) | 674 (91.1%) |
| **Disc prolapse** | | | | |
| Yes | 82 (3.7%) | 13 (4.9%) | 117 (19.5%) | 106 (14.3%) |
| No | 2147 (96.3%) | 254 (95.1%) | 484 (80.5%) | 634 (85.7%) |
| **Dysesthesia (tingling)** | | | | |
| Yes | 174 (7.8%) | 26 (9.7%) | 130 (21.6%) | 136 (18.4%) |
| No | 2055 (92.2%) | 241 (90.3%) | 471 (78.4%) | 604 (81.6%) |
| **Back pain in last 3 month (NRS)** | | | | |
| Mean (SD) | 2.07 (2.38) | 2.56 (2.38) | 3.79 (2.68) | 3.76 (2.81) |
| **Back pain irradiation (ref: no)** | | | | |
| no | 1761 (79.0%) | 190 (71.2%) | 281 (46.8%) | 393 (53.1%) |
| gluteal only | 274 (12.3%) | 40 (15.0%) | 144 (24.0%) | 146 (19.7%) |
| to knee | 136 (6.1%) | 21 (7.9%) | 102 (17.0%) | 114 (15.4%) |
| to lower leg | 58 (2.6%) | 16 (6.0%) | 74 (12.3%) | 87 (11.8%) |
| **Medication (NSAIDs, Opioids, Benzodiazepine)** | | | | |
| Yes | 183 (8.2%) | 26 (9.7%) | 118 (19.6%) | 151 (20.4%) |
| No | 2046 (91.8%) | 241 (90.3%) | 483 (80.4%) | 589 (79.6%) |
| **Physician visit (last 4 weeks, self-reported)** | | | | |
| No physician visit | 567 (25.4%) | 50 (18.7%) | 43 (7.2%) | 57 (7.7%) |
| GP visit only | 1381 (62.0%) | 169 (63.3%) | 275 (45.8%) | 358 (48.4%) |
| Specialist only | 42 (1.9%) | 5 (1.9%) | 43 (7.2%) | 44 (5.9%) |
| GP and specialist | 239 (10.7%) | 43 (16.1%) | 240 (39.9%) | 281 (38.0%) |

*Seek of care:
0|0 = no ICD-10 codes suggestive for LBP in any of the analysis periods,
0|1 = ICD-10 codes suggestive for LBP only during follow-up,
1|1 = ICD-10 codes suggestive for LBP in all analysis periods,
1|0 = history of back pain prior baseline and/or ICD-10 codes suggestive for LBP at baseline but not in the follow-up

## References


1. Sundararajan V, Henderson T, Perry C, Muggivan A, Quan H, Ghali WA. New ICD-10 version of the charlson comorbidity index predicted in-hospital mortality. Journal of clinical epidemiology. Elsevier; 2004;57:1288–94.

2. Wood SN. Stable and efficient multiple smoothing parameter estimation for generalized additive models. Journal of the American Statistical Association. Taylor & Francis; 2004;99:673–86.

3. Richter A, Schmidt CO, Krüger M, Struckmann S. dataquieR: Assessment of data quality in epidemiological research. Journal of Open Source Software [Internet]. The Open Journal; 2021;6:3093. Available from: https://doi.org/10.21105/joss.03093

4. Mayr A, Hofner B, Waldmann E, Hepp T, Meyer S, Gefeller O. An update on statistical boosting in biomedicine. Computational and mathematical methods in medicine. Hindawi; 2017;2017.

5. Hothorn T, Buehlmann P, Kneib T, Schmid M, Hofner B. mboost: Model-based boosting [Internet]. 2020. Available from: https://CRAN.R-project.org/package=mboost

6. Hofner B, Boccuto L, Göker M. Controlling false discoveries in high-dimensional situations: Boosting with stability selection. BMC bioinformatics. Springer; 2015;16:1–7.

7. Hastie T, Tibshirani R, Friedman J. The elements of statistical learning: Data mining, inference, and prediction. Springer Science & Business Media; 2009.

8. Steyerberg EW, Harrell Jr FE. Prediction models need appropriate internal, internal-external, and external validation. Journal of clinical epidemiology. NIH Public Access; 2016;69:245.

9. Filzmoser P, Liebmann B, Varmuza K. Repeated double cross validation. Journal of Chemometrics [Internet]. 2009;23:160–71. Available from: https://analyticalsciencejournals.onlinelibrary.wiley.com/doi/abs/10.1002/cem.1225

10. Dawid AP, Sebastiani P. Coherent dispersion criteria for optimal experimental design. The Annals of Statistics [Internet]. Institute of Mathematical Statistics; 1999;27:65–81. Available from: http://www.jstor.org/stable/120118

11. Gneiting T, Raftery AE. Strictly proper scoring rules, prediction, and estimation. Journal of the American Statistical Association [Internet]. Taylor & Francis; 2007;102:359–78. Available from: https://doi.org/10.1198/016214506000001437

12. Wei W, Held L. Calibration tests for count data. TEST [Internet]. 2014;23:787–805. Available from: https://doi.org/10.1007/s11749-014-0380-8

13. Meyer S, Held L, Höhle M. Spatio-temporal analysis of epidemic phenomena using the r package surveillance. Journal of Statistical Software, Articles [Internet]. 2017;77:1–55. Available from: https://www.jstatsoft.org/v077/i11

14. Corporation M, Weston S. doParallel: Foreach parallel adaptor for the 'parallel' package [Internet]. 2020. Available from: https://CRAN.R-project.org/package=doParallel

15. University of Greifswald. HPC brain cluster [Internet]. Available from: https://rz.uni-greifswald.de/dienste/allgemein/sonstiges/high-performance-computing/

16. Czado C, Gneiting T, Held L. Predictive model assessment for count data. Biometrics [Internet]. 2009;65:1254–61. Available from: https://onlinelibrary.wiley.com/doi/abs/10.1111/j.1541-0420.2009.01191.x

17. van Buuren S, Groothuis-Oudshoorn K. mice: Multivariate imputation by chained equations in r. Journal of Statistical Software [Internet]. 2011;45:1–67. Available from: https://www.jstatsoft.org/v45/i03/

18. Rubin DB. Multiple imputation after 18+ years. Journal of the American statistical Association. Taylor & Francis Group; 1996;91:473–89.

19. Jakobsen JC, Gluud C, Wetterslev J, Winkel P. When and how should multiple imputation be used for handling missing data in randomised clinical trials–a practical guide with flowcharts. BMC medical research methodology. BioMed Central; 2017;17:1–0.

20. Breiman L. Random forests. Machine learning. 2001;45:5–32.

21. Biau G, Scornet E. A random forest guided tour. Test. Springer; 2016;25:197–227.

22. Probst P, Wright MN, Boulesteix A-L. Hyperparameters and tuning strategies for random forest. WIREs Data Mining and Knowledge Discovery [Internet]. 2019;9:e1301. Available from: https://wires.onlinelibrary.wiley.com/doi/abs/10.1002/widm.1301

23. Kleiber C, Zeileis A. Visualizing count data regressions using rootograms. The American Statistician. 2016;70:296–303.
